# Author index to Volume 81

**DOI:** 10.1038/sj.bjc.6690864

**Published:** 1999-12


					Aaronson NK 87
Abbruzzese A 1134
Abd Alsamad I 1344
Abe T 469
Abel F 1402
Abeler VM 75
Abenhaim L 62
Ablashi D 893
Abolafio G 252
Abraham J 520
Abrahamson J 179
Adami H-O 159
Adams GE 616
Adlercreutz H 574 (A)
Adrover E 122
Ahn Y 1116
Al-Nabulsi I 925
Alderson D 571 (A)
Alexopoulos CG 69
Alhava EM 133
Allen-Mersh TG 572 (A)
Amadori D 609
Ames BN 566 (A)
Ammerman CA 1328
Andersen EW 907
Anderson VA 141
Andersson J 359
Andersson LC 1017
Andersson U 359
Ando M 1419
Andrew N 573 (A)
Annink C 141
Ansari B 574 (A)
Anwar S 571 (A), 572 (A)
Aoki S 108
Aparicio T 1351
Appleby PN 1248
Archer C 316
Ardissone F 841
Ardizzoni A 310
Armand J-P 457
Armatis P 966
Arrand JE 377
Arribas GLB 1238
Arzt E 381
Astier-Gin T 860
Atherton K 574 (A)
Atkin SL 690
Audhuy B 449
Austin S 1162
Austyn JM 1280
Axdorph U 1182
Babb J 1222
Bacci F 609
Badiali De Giorgi L 404
Bahl P 1280
Bailey AJ 1196
Bain CJ 559
Bakker AQ 43
Balesaria S 1371
Balibrea JL 122
Ballard-Barbash R 167
Bamber D 572 (A)
Bamber DE 571 (A)
Bandoh S 769
Barbarulo D 1134
Barber MD 80
Bardou VJ 449
Barnes D 576 (A)
Barragan M 294
Bartrons R 294
Bartsch G 242
Barzanti F 609
Bates S 565 (A)
Batra V 1022
Battista Ratto G 310
Baudin E 1351
Baum M 1427
Bawden LJ 19
Beale P 1022
Bearzatto A 252
Beemsterboer PMM 912
Begent RHJ 972
Begin LR 850
Beijnen JH 330
Beitner R 219
Belitchenko I 37
Bellagamba O 1378
Belzile E 62
Benedetti-Panici P 733
Bengtsson E 1363
Beppu K 721
Beral V 1248
Bergstrom R 159
Berkel HJ 1
Bernard I 1344
Berndt A 1071
Berns MW 631
Bernstein L 532
Berruti A 841
Bertheau P 832
Bertolazzi L 1378
Bertolini F 1398
Beverley E 554
Bezdetnaya L 37
Bibby M 583 (A)
Bibby MC 367, 1318
Biberfeld P 1182
Bicknell EJ 571 (A)
Biggar R 893
Bijleveld EH 1150
Bingham S 568 (A), 578 (A)
Birch JM 300
Bjork J 232
Bjorkholm M 1182
Bjorkqvist A-M 1111
Blok LJ 28
Blomqvist CP 1017
Bode MK 654
Boer R 912
Bohling TO 1017
Bohm W 702
Boivin J-F 62
Bokemeyer C 1052
Bone EA 19
Bonetti A 1213
Boni L 310
Bonichon F 860
Bonilla F 503
Bonoron-Adele S 24
Borasio P 841
Bordessoule S 457
Borg A 672
Borsi L 1071
Boshoff C 893
Bosman FT 934
Bouchier-Hayes DJ 1311
Bouvier V 305
Bower M 316, 1371
Bowey EA 574 (A)
Brada M 1022
Bradley JK 616
Brain Tumours Subcommittee of
the French Society of
Paediatric Oncology (SFOP)
835
Branigan K 1174
Brannigan JA 576 (A)
Brash M 409
Bratt O 672
Breidenbach M 1165
Breitenecker G 855
Bremner JCM 616
Brent TP 576 (A)
Bresaola E 1213
Bright RK 54
Brimmell M 1042
Brinkmann AO 28
Brinton LA 167
British Stomach Cancer Group
1356
Brizzi MP 841
Brock C 1371
Brodie AMH 622
Broeders MJM 912
Brogan DR 167
Brown J 580 (A)
Brown K 577 (A)
Brown KM 638
Brown NJ 287
Brown SB 616
Broxterman HJ 269
Brunet J-S 179, 850
Brunner N 203
Buchan S 580 (A)
Buckley JD 900
Budach V 1206
Budillon A 1134
Buffa R 1398
Bui TD 496
Bulzebruck H 510
Burger AM 367
Burgess M 583 (A)
Burns JS 1042
Burns P 567 (A)
Burtles SS 760
Burton A 1356
Busch C 1363
Buxbaum P 662
Cabria M 1378
Calautti E 756
Callaghan R 783
Cameron IL 440
Campbell FC 570 (A), 574 (A)
Campioni N 1031
Cann SA 1426 (L)
Cao J 342
Caraglia M 1134
Carbone M 54
Carboni N 1398
Carles P 1059
Carmichael PL 566 (A)
Carthew P 570 (A), 579 (A)
Cartwright R 1228
Casas A 13
Case MC 576 (A)
Casey JL 972
Cassidy J 99
Castano E 294
Castelao JE 542
Cazap E 846
Cerdan J 122
Cetto GL 1213
Chan JYH 994
Chan W-K 554
Chandana AK 893
Chang GTG 28
Chao D 1280
Chapman P 575 (A)
CHART Steering Committee
1196
Chastagner P 835
Chatlynne L 893
Chen C-J 537
Chen J 814, 994
Chen J-C 796
Chen Y-C 796
Chern H-D 537
Cheung ST 1122
Chi C-W 416
Author index to Volume 81
Key to abbreviations:
(A) Abstracts; (BR) Book reviews; (L) Letters to the editors
1429
British Journal of Cancer (1999) 81(8), 1429-1435
(c) 1999 Cancer Research Campaign
Article no. bjoc.1999.0864
Childers SR 925
Chinje EC 1127
Chiou H-Y 537
Choi K-Y 1116
Chouaki N 457
Chouffai Z 835
Christian W 1052
Chung NYF 1122
Churdboonchart V 893
Cinat G 846
Clark GM 490
Clark J 1371
Clark JW 1002
Clarke K 152
Clarke P 565 (A)
Claudiani F 1378
Cleghorn F 893
Clerc X 800
Cleton-Jansen A-M 1410
Coates RJ 167
Cognetti F 1031
Cohen BB 141
Colditz GA 918
Cole DJ 1002
Coleman RE 1037
Collet J-P 62
Collins HM 19
Coltman CA 184 (L)
Connor JM 573 (A)
Connors TA 760
Coombes RC 316
Cooper H 893
Cooper RA 354
Cornelisse CJ 1410
Cornelissen IMHA 774
Cortelezzi A 1398
Cortesse A 832
Cosso M 310
Costa B 449
Cote RJ 184 (L)
Couanet D 835
Coughtrie MWH 571 (A)
Coupe A 583 (A)
Couteau C 457
Coutier S 37
Coutts J 571 (A)
Cozen W 532
Craft AW 336
Creighton AM 800
Crew T 568 (A)
Cruz I 881
Culig Z 242
Cure H 449
Cutler D 1022
Cuzick J 554
Czarnota GJ 520
Da Costa M 1311
Daidone MG 252
Dal Cortivo L 832
Dal Susino M 609
Daling JR 167
Dalmau M 294
Daly M 1222
Davidson SE 354
Davies MJ 574 (A)
Davis W 566 (A)
Day N 1257
de Bernardi B 1378
de Both NJ 934
de Bruyn AE 912
De Forni M 457
de Gast GC 43
de Jong MC 269
de Koning HJ 912
De Marco C 252
De Pasqua A 733
De Pont JJHHM 28
de Sanctis S 1371
de Vries TJ 1066
Deag E 578 (A)
Deakin M 572 (A)
Debled M 860
Deeg HJ 808
del Barco V 122
del C Batlle AM 13
Del Vecchio MR 1031
Delozier T 449
Delva R 449
Demaine AG 573 (A), 580 (A)
Denkena A 1009
Denman AR 1243
Dent DM 1142
Derenzini M 404
Desgrandchamps F 832
Detmar SB 87
Devaja O 582 (A)
Devauchelle P 1344
Devereux S 476
Dhar DK 712
Dhar KK 1174
Di Gennaro E 1134
Di Stefano G 404
Diamandis E 1222
Diamandis EP 490
Dickinson HO 144
Diepstra H 1066
Dietel M 790
Dinh A 575 (A)
Dinjens WN 934
Dix RK 24
Dixon JM 1088
Dobashi N 769
Dogliotti L 841
Dohlsten M 359
Doireau V 835
Dolan K 581 (A)
Dolcetti A 841
Doll R 3
Domenge C 457
Dominguez G 503
Dono K 747
Dorbic T 1009
Dosaka-Akita H 696
Double JA 367
Drivsholm L 667
Du W 54
Duan R-D 232
Ducreux M 1351
Duffy S 1257
Dugan M 1022
Duncan R 577 (A), 583 (A)
Dunn J 581 (A)
Dunn JA 1356
Duvillard P 1351
Earl H 187 (BR)
Eberhardt W 1206
Eder IE 242
Edwards DM 323
Edwards J 893
Edwards RE 570 (A), 579 (A)
Eeles RA 1099
Eghbali H 860
Egilsson V 1103
Ehya H 1222
Einatan J 874
Eiriksdottir G 1103
Ejeskar K 1402
Ekman M 1182
Elder DJE 568 (A)
Elder J 571 (A), 572 (A)
Elder JB 571 (A)
Elfving P 6
Elias D 1351
Elnatan J 237
Embleton MJ 577 (A)
Engel H 1165
English J 316
English MW 336
Engstrom PF 1222
Enns L 959
Entezami Z 581 (A)
Epenetos AA 431
Erdel M 242
Eskelinen MJ 133
Espana P 503
Ethier SP 1328
Evans H 1371
Fabbro M 449
Fadl-Elmula I 6
Fagotti A 733
Faivre J 463
Farrow S 575 (A)
Fearon KCH 80
Fehr MK 631
Feldmann KA 572 (A)
Felix D 580 (A)
Fenhalls G 1142
Fernandez C 122
Ferrandina G 733
Ferraresi V 1031
Ferrero JM 449
Ferrucci PF 1398
Ferry DR 783, 942
Feskanich D 918
Feuilhade F 449
Fidler IJ 647
Field JK 581 (A)
Fielding JWL 1356
Filion MC 584 (A)
Finke S 1009
Fischer H 190
Fisher RA 1371
Fiume L 404
Flasshove M 1206
Fleetwood A 571 (A)
Fleury-Feith J 1344
Folli S 609
Foppiano F 310
Ford AM 898
Forozan F 1328
Foulkes WD 850
Fourkour A 1066
Fox BW 760
Fracheboud J 912
Fraggetta F 1213
Fraier D 99
Franceschi S 890
Frassineti GL 609
Freshney RI 924 (BR)
Friedrich J 1165
Frierson Jr HF 75
Frigerio E 99
Fryer A 572 (A)
Fryer AA 571 (A), 1174
Fujii H 1419
Fujimoto Y 393
Fujita J 769
Fujiwara K 1080
Fujiyoshi K 95
Fukuda H 13
Fukumura D 756
Furui J 350
Gago-Dominguez M 542
Gallagher JT 571 (A)
Gallina A 567 (A)
Gallus S 890
Gamble SC 377
Gammon MD 167
Garau I 463
Garaventa A 1378
Garbett EA 287
Garcia JM 503
Garde J 581 (A)
Gastl G 185 (L)
Gatehouse D 578 (A)
Gerbino A 841
Gerritsen WR 43
Gescher A 568 (A), 575 (A)
Geyp M 1142
Gianasi E 577 (A)
Gibson I 582 (A)
Gignoux M 305
Gil J 294
Gill P 893
Gilligan D 187 (BR)
Gillissen RAHJ 571 (A)
Giudici S 310
Glaser MG 1371
Glass-Marmor L 219
Glatt HR 566 (A)
Glimelius B 190
Glover DW 1426 (L)
Godwin A 179
Goh H-S 237, 874
Goldstone AH 476
Gonzalez R 503
Goodman MT 1248
Gordon PH 850
Gorunova L 6
Gorzegno G 841
Gosney J 581 (A)
Grankvist K 727
Gray AM 1243
Greaves M 898
Greb RR 225
Green AC 559
Green AJ 972
Gregory W 476
Greschuchna D 1206
Gridley G 175
Griech E 578 (A)
Grieshaber CK 753
Griffiths GO 1196
Griffiths J 616
Grignon Y 835
Grigoryev DN 622
1430 Author index
British Journal of Cancer (1999) 81(8), 1429-1435 (c) Cancer Research Campaign 1999
Grill J 835
Grimfors G 1182
Grosios K 1318
Grossi F 310
Groult V 457
Groves FD 175
Guillemin F 37
Guillot A 449
Gullu IH 186 (L)
Gunsilius E 185 (L)
Gurney JG 549
Haak H 684
Hacker NF 559
Hague A 568 (A)
Hall AJ 1097
Hall HI 167
Hall M-C 690
Hallissey MT 1356
Halls SB 638
Hamblin MR 261
Hamdy FC 832
Hamel BC 1150
Hamers MJAG 203
Han X 582 (A)
Hancock A 583 (A)
Hancock B 476
Hancock BW 1037
Hand P 1174
Hanigan MH 75
Hanley J 62
Haque K 584 (A)
Hara K 705
Harada N 1155
Hardman WE 440
Harima K 108
Harima Y 108
Harmey JH 1311
Harnden DG 189
Harper-Wynne C 316
Harris AL 496, 1127, 1280
Harrison CN 476
Harrison K 584 (A)
Harrison KL 584 (A)
Harrison PR 567 (A), 580 (A)
Harstrick A 1304
Hartmann J 1052
Hartung G 1428 (L)
Haruma K 647
Hasan T 261
Hata E 705
Hatano K 1274
Hatzidimitriou G 69
Hawkins K 19
Hayakawa M 277
Hayashi H 469
Hayes JD 567 (A)
Hazama S 469
He JH 994
Hedley DW 989
Heemskerk MHM 43
Heene DL 1428 (L)
Hefler L 855
Heidtmann H-H 1269
Heijn M 269
Heim S 6
Heinze G 855
Heinzl H 662
Hekman A 43
Helmerhorst ThJM 114
Hendriks JHCL 912
Hendry JH 577 (A)
Henriksson R 727
Herbert C 305
Hermsen MA 1150
Hertervig E 232
Heuft H-G 1009
Hewer A 566 (A), 570 (A)
Heydon R 577 (A)
Heydon RT 579 (A)
Hg NK 994
Higashi T 1080
Higgins CF 783
Hilger RA 1304
Hill BT 800
Hilton D 580 (A)
Hirai Y 1234
Hirao Y 741
Hirose K 469
Hiroumi H 696
Hisada T 705
Hiscox S 579 (A)
Hishikawa Y 712
Hittmair A 242
Hiura M 95
Hjelm NM 1188
Hjerl K 907
Ho CS 1188
Ho L 554
Ho SKW 1188
Hoban PR 1174
Hobisch A 242
Hoekstra HJ 1262
Hoerni B 860
Hoffman J 1222
Hoffmann J 242
Hofmann UB 774
Holland T 571 (A)
Holloway KA 575 (A)
Hollowood AD 571 (A)
Holly JMP 571 (A)
Holst-Hansen C 203
Holwell S 1318
Hommura F 696
Hooijberg JH 269
Hoon DSB 342
Hooper ML 141
Hopfner U 381
Hornung R 631
Hoskin P 476
Hostein I 860
Houlbrook S 1280
Howells L 575 (A)
Howells REJ 1174
Hoy L 1280
Hradil K 1052
Hsieh F-I 537
Hsu L-I 537
Huang DP 1122
Hubbard A 575 (A)
Hudson A 575 (A)
Hudson EA 568 (A), 575 (A)
Hudson M 431
Hughes R 578 (A)
Huhn D 1009
Huiping C 1103
Huizing MT 330
Hulsebos TJM 1150
Hultkrantz R 232
Hunt JW 520
Hunter DJ 918
Hunter RD 354
Huss R 808
Huuhtanen RL 1017
Hyckel P 1071
Iannucci A 1213
Ichinose K 350
Igarashi K 705
Iizuka N 469
Ikeguchi M 484
Ikematsu Y 350
Ikuta K 393
Ingvarsson S 1103
Inoue K 350
Irie RF 342
Ishihara S 1274
Ishizaki M 1080
Ito Y 747
Iversen T 528
Jack N 893
Jackson RT 1238
Jacobs MV 114
Jacquemier J 449
Jagdev S 1037
Jager R 1269
Jain RK 756
James LA 300
Janot F 457
Jansen CT 1426
Janvier M 449
Jarrett RF 898
Jaurand MC 1344
Jayson GC 571 (A)
Jenkins TC 367
Jensen F 893
Jiang WG 579 (A)
Jodrell DI 760
Joel SP 1285
Johannessen BE 203
Johannsdottir JT 1103
Johnson NW 323
Johnson PJ 1188
Jonas SK 572 (A)
Jonas U 1052
Jonasson JG 1103
Jones P 572 (A)
Jorgensen T 907
Judson I 1022
Jukes R 577 (A)
Jung R 870
Kaern J 75
Kaibara N 484
Kainz C 855
Kajiyama G 647
Kaklamanis L 496
Kalemkerian GP 54
Kalifa C 835
Kallenborn A 1165
Kallioniemi A 1328
Kallioniemi O-P 1328
Kalman E 510
Kanazawa K 1234
Kane EV 1228
Kanematsu T 350
Kaneyoshi T 1080
Kaprio J 1426
Kaptein A 575 (A)
Karaaslan S 790
Kariyama K 1080
Kashii Y 822
Katenkamp D 1071
Katoh H 696
Katsumata N 1419
Kauppila S 654
Kawakami Y 696
Kawana T 1234
Kay E 1311
Kayser K 510
Keiding N 907
Keith WN 684
Kelland LR 1294
Kellett GL 423
Kellokoski JK 133
Kelly B 582 (A)
Kelly S 573 (A)
Kelsey AM 300
Keng PC 925
Kennedy CA 1243
Kere J 1111
Kerr DJ 942, 1356
Keum JS 127
Key TJ 1248
Kheuang L 1344
Kiaris H 966
Kieback DG 179
Kiesel L 225
Kim H 1116
Kim JJ 1116
Kim N-G 1116
King DJ 972
Kinoshita I 696
Kinugasa S 712
Kitadai Y 647
Kitayama J 1274
Klaassen U 1304
Kleespies C 1165
Klein-Szanto A 1222
Klocker H 242
Klungland A 576 (A)
Knuchel R 1052
Knuutila S 1111
Knuuttila A 1111
Kobayashi Y 1080
Koberle B 1294
Kogner P 1402
Kohgo Y 393
Kohlberger P 702
Koizumi K 1155
Koji T 712
Kojima M 721
Kokubun T 575 (A)
Kolhagen H 1165
Kolios MC 520
Kolks S 1206
Kondo A 484
Kong G 127
Kontermann RE 1269
Korte M 1009
Kos J 510
Koskenvuo M 1426
Kosma V-M 133
Kosmehl H 1071
Kote-Jarai Z 1099
Koumarianou AA 431
Kowalski LP 677
Krasovec M 510
Krausz T 554
Author index 1431
British Journal of Cancer (1999) 81(8), 1429-1435
(c) Cancer Research Campaign 1999
Kreienberg R 179, 702
Kressner U 190
Kristensen CA 756
Kristoffersson U 672
Kruger W 870
Kubba A 574 (A)
Kubbutat M 565 (A)
Kuczyk M 1052
Kulphadist SAR 893
Kumagai S 95
Kunkler IH 141
Kuo M-L 796
Kurdoglu M 186 (L)
Kurian KM 141, 1088
Kuroki T 350
La Vecchia C 890
Labat JP 449
Lah TT 510
Lai T 571 (A)
Lakhani SR 1042
Lambertenghi-Deliliers G 1398
Langman MJS 1356
Lanino E 1378
Lankelma J 269
Lanyon WG 573 (A)
Lasser P 1351
Launoy G 305
Lazarevic B 981
Le Duc A 832
Le Pimpec-Barthes F 1344
Leanderson T 981
Lebecq A 457
Lee JCK 1122
Lee JD 127
Lee JH 127
Lee Y-S 237
Lefevre H 305
Lefterova P 1009
Lellouch-Tubiana A 835
Leodolter S 855
Lerman C 179
Leung LK 387
Leung SF 994
Leuratti C 578 (A)
Levesque MA 490
Levine PH 893
Lewis EL 582 (A)
Lewis P 1371
Leyland-Jones B 753
Leyvraz S 457
Liberg D 981
Liegmann K 893
Lim SH 1162
Lin H-L 416
Lin ZY 814
Linassier C 449
Linch DC 476
Lindahl T 576 (A)
Lindblom A 190
Linderholm B 727
Lindmark G 190
Lindtner B 1304
Linet MS 175
Lipponen PK 133
Litton MJ 359
Liu T-Y 416
Liu Y 622
Liu YK 814
Lo KW 1122
Lo Piccolo MS 1378
Lockyer SD 1127
Logue JP 354
Long BJ 622
Lord BI 1095 (L)
Lorigan PC 1037
Lorincz A 554
Lorrimore SA 570 (A)
Lotterle H 510
Low PB 573 (A)
Lowdell C 316
Luben R 1257
Lucas LT 578 (A)
Ludwig R 565 (A)
Lundgren R 6, 672
Mabuchi K 1248
McCallum TJ 638
McCann J 1257
McCulloch PG 1392
McDonald L 1042
Macey M 581 (A)
McFadyen MCE 580 (A)
McGown AT 1318
McGregor F 580 (A)
Mach RH 925
Machtens S 1052
Mack TM 532
MacKenzie J 898
MacKie RM 573 (A)
MacMillan AK 476
McNicol AM 684
Maestro ML 122
Maeta M 484
Maier I 225
Maitre N 1002
Major GN 576 (A)
Makimoto H 1155
Malick J 1222
Malik N 577 (A)
Mallmann P 1165
Malmstrom P-U 1363
Malone KE 167
Mancuso S 733
Mandahl N 6
Mandel U 1071
Maneschi F 733
Manning LS 952
Manns A 893
Mansel RE 579 (A)
Manson MM 567 (A), 568 (A),
575 (A)
Mao Y 152
Marchal JC 835
Marchal S 37
Margison GP 565 (A), 577 (A),
584 (A)
Marnett LJ 578 (A)
Marolleau J-P 832
Marson LP 1088
Martin EA 577 (A), 579 (A)
Martinez G 503
Martinsson T 1402
Masaki T 705
Masood R 893
Masters JRW 1294
Matsui J 1155
Matsumoto K 194, 721
Matsumoto-Taniura N 194
Matsunari Y 721
Matsuura N 747
Mattson K 1111
Mauduyt M-A 1059
Maurel J 305
Mayerhofer K 855
Mayumi T 1155
Medl M 662
Mee T 212
Meijer CJLM 114, 881
Melder RJ 756
Melnikova V 37
Melvin WT 580 (A)
Menard I 584 (A)
Mendoza N 1371
Mereu C 310
Merlin JL 37
Metcalfe S 212
Meyer L 316
Mezzelani A 586
Miedouge M 1059
Mielzynska I 554
Migaldi M 404
Milan T 1426
Milanaccio C 1378
Milella M 1031
Miller GG 638
Miller ID 580 (A)
Miller WR 1088
Milligan D 476
Milner J 212, 409
Mingels A 1269
Minoletti F 586
Miracca EC 677
Miranda N 43
Mirzayans R 959
Mishina T 696
Missale C 381
Mitelman F 6
Mitry E 1351
Mizumoto K 721
Molina A 600
Momma T 261
Monden M 747
Monnet E 463
Monti-Frayne J 631
Moody PCE 576 (A)
Moore RB 638
Moore S 1022
Moreno-Merlo F 989
Morgan G 564 (BR), 1228
Morisaki T 721
Morris TM 19
Mortensen PB 907
Moschini T 841
Moslehi R 179
Mothersill CE 570 (A)
Mott M 583 (A)
Mottolese M 733
Mousseau M 449
Moyer MP 440
Mukaida N 647
Muller EJ 43
Muller MR 1206, 1304
Munks RJL 575 (A)
Munson P 1042
Murai M 277
Murphy D 874
Murray D 959
Murray GI 580 (A)
Murray LS 99
Musgrove C 1174
Muto T 1274
Nagai MA 677
Nagano H 747
Nagasue N 712
Nagata C 1234
Nagata T 705
Nagawa H 1274
Nagy A 966
Naka K 647
Nakagawa Y 741
Nakahara M 747
Nakajima J 705
Nakamori S 747
Nakamura H 277
Nakamura T 194, 721
Nakao K 747
Nakatsuka A 721
Nakatsukasa H 1080
Nakayama E 1080
Nannan Panday VR 330
Nardi M 1031
Narod SA 179
Nash J 1392
Natali PG 733
Neal GE 567 (A)
Neglia JP 175
Negri E 890
Nettelbeck DM 1269
Neubauer A 1009
Neumaier M 870
Newcomb PV 571 (A)
Newell DR 760
Newland AC 476
Newlands ES 1371
Nicholson KM 423
Nicholson SK 1371
Nicklee T 989
Nilsen H 576 (A)
Nilsson A 232
Nishida T 95
Nishio S 95
Nita ME 1274
Niwa T 1419
Njar VCO 622
Nnane IP 622
Noda K 1234
Nolan BM 570 (A), 579 (A)
Noma T 469
Nooijen WJ 330
Nordling S 1111
Norhanom A 893
Norrish AE 1238
Notarianni GB 576 (A)
Nouri AME 581 (A)
Nouso K 1080
Nozawa S 1234
Obermair A 662
O'Connor PJ 565 (A)
Ogura S 696
O'Hare MJ 1042
Ohhira M 393
Ohizumi I 1155
Ohlsson L 359
Ohmoto Y 647
Ohnishi T 108, 1080
Ohsugi Y 1155
Ohta M 1419
1432 Author index
British Journal of Cancer (1999) 81(8), 1429-1435 (c) Cancer Research Campaign 1999
Ohta Y 54
Ohtake T 393
Ohtsuki Y 769
Oka M 469
Oka S 484
Oka T 705
Okorokov AL 212
Okumura A 1419
Okura N 95
Olipade O 179
Oliver RTD 581 (A)
Olsen JH 907
Olsson H 672
Omata M 705
Omuro Y 1419
O'Neill CF 1294
Ono M 393
Oosterwijk E 741
O'Prey J 580 (A)
Orlandi L 252
Orlando M 846
Orr FW 1002
Osada T 1274
Osborne MR 566 (A)
Oskam NC 1150
Osterlind K 667
Ottensmeier C 1213
Ottensmeyer FP 520
Oulid-Aissa D 457
Owen-Jones E 1162
Paakko P 592
Packham G 1042
Paez Pereda M 381
Pagotto U 381
Pahlman L 190
Palangie T 449
Paloheimo LI 667
Pang JCS 994
Paraskeva C 568 (A), 571 (A)
Parczyk K 242
Parekh R 1188
Park K-S 1116
Park SS 127
Parker J 1228
Parker L 144
Parker MI 1142
Parkes J 1174
Parmar MKB 1196
Parney I 638
Parsa NZ 1328
Pasini F 1213
Pasquali-Lasagni R 1031
Pass HI 54
Patel T 1188
Pateras C 1385
Patnaik M 893
Patterson AV 1127
Pavanel F 1213
Payne S 580 (A)
Pearson ADJ 336
Pedley RB 972
Pelosi G 1213
Penso J 219
Perks CM 571 (A)
Pero RW 981
Perry J 898
Peters G 565 (A)
Peters-Engl C 662
Peterse JL 1410
Petersen K 870
Peterson D 1371
Pettit GR 1318
Pezzella F 1398
Phillip A 565 (A)
Phillips DH 566 (A), 570 (A)
Phillips HE 584 (A)
Phillips NC 584 (A)
Phillips PS 1243
Pidgeon GP 1311
Pierotti MA 586
Pietilainen T 133
Pike MC 184 (L)
Pilotti S 586
Pinedo HM 269
Pinsky L 850
Platel D 24
Plummer SM 568 (A), 575 (A)
Pollock BH 549
Poon TCW 1188
Portnoy M 520
Porwit-MacDonald A 1182
Potischman N 167
Potter PM 576 (A)
Pouillart P 449
Poulsom R 496
Povey AC 565 (A), 584 (A)
Prange G 1009
Press MF 532
Price L 336
Provencio M 503
Pruneri G 1398
Pu Y-S 537
Pueyo G 294
Pujazon M-C 1059
Punt CJA 1066
Purdie DM 559
Quinn DM 423
Quinn MA 559
Quintin Colonna F 1344
Rabkin C 893
Radin NS 952
Rajadhyaksha M 261
Raju K 582 (A)
Ramesh S 1392
Ranefall P 1363
Raymond L 463
Redfern A 579 (A)
Redman CWE 1174
Redmond HP 1311
Reed MWR 287
Regele S 702
Reichert TE 822
Reidenberg P 1022
Reinthaller A 855
Reitz M 893
Rengifo W 705
Renier A 1344
Renner U 381
Reyre J 1059
Richardson S 577 (A)
Ricotti L 609
Riquet M 1344
Risch H 179
Risteli J 654
Risteli L 654
Roberge S 756
Robert J 24
Roberts SA 354
Robinson L 1162
Robison LL 175, 549, 900
Roche H 449
Rodriguez Nievas O 846
Roman E 1228
Roper DI 576 (A)
Ropponen KM 133
Rosenberg C 1410
Rosendahl A 359
Ross JA 80, 549
Ross RK 184 (L), 542
Ross SJ 576 (A)
Rosso R 310
Rougier P 1351
Rousseau F 449
Rousselle P 1071
Rouzaud P 1059
Rovers JP 600
Rowland IR 574 (A)
Rufie P 1351
Ruiter DJ 774, 1066
Rumsby PC 574 (A)
Runnebaum IB 179, 702
Russell AJ 684
Russell P 559
Rustum YM 1304
Saarnak AE 600
Sabourin J-C 1351
Sack H 1206
Saga S 741
Saito H 393, 484, 1155
Sakon M 747
Sala E 1257
Salahshor S 190
Salama G 1059
Salerno MG 733
Sandler DP 900
Santomaggio C 1031
Sanz-Casla MT 122
Sapper H 554
Sasaki Y 1419
Sat YN 577 (A), 583 (A)
Satchi R 577 (A)
Sattar A 570 (A)
Saunders MP 1127
Sauter ER 1222
Sawitz A 907
Scambia G 733
Schaapveld M 1262
Schally AV 966
Schanzenbacher U 510
Scheffer GL 269
Schellens JHM 330
Scheper RJ 269
Schirren J 510
Schlenger L 1206
Schleucher N 1304
Schlumberger M 1351
Schmidt-Wolf GH 1009
Schneider MR 242
Schoenberg JB 167
Schofield K 571 (A)
Schondorf T 1165
Schornagel JH 87
Schraffordt Koops H 1262
Schubach J 1052
Schuitmaker JJ 600
Schutte J 1206
Schwella N 1009
Scinto AF 1031
Scolaro T 310
Scopinaro G 1378
Scorilas A 1385
Seeber S 1206, 1304
Seedhouse CH 577 (A)
Sekiya S 1234
Selby PB 1094 (L)
Sena S 846
Serre G 1059
Serth J 1052
Sevelda P 662
Severson RK 549
Seymour CB 570 (A)
Sharma S 1037
Sharp GB 1248
Sharpe C 62
Sharpe SJ 1238
Sherar MD 520
Shibata Y 1274
Shields PG 567 (A)
Shimizu H 1234
Shimizu N 277
Shin DH 127
Shirahama S 108
Shiratori Y 705
Shiu RPC 1002
Shridhar V 54
Shu XO 175
Shuker DEG 578 (A), 584 (A)
Sibbald J 684
Sigurdson E 1222
Sigurgeirsdottir JR 1103
Sijmons RH 1262
Silva JM 503
Simmonds M 575 (A)
Singh R 578 (A)
Sinn H 1428 (L)
Sinnett HD 316
Siskind V 559
Sjoberg J 1182
Skeaff CM 1238
Skinner E 184 (L)
Skinner R 336
Sleijfer DTh 1262
Smith DR 237, 874
Smith K 496
Smith LL 565 (A), 570 (A), 579
(A)
Smith PG 1097
Sneeuw KCA 87
Snell L 1002
Snijders PJF 114, 881
Soda M 1248
Sogaard M 359
Soini Y 592
Soler M 890
Soligo D 1398
Sondergaard KL 573 (A), 580
(A)
Sonneveld DJA 1262
Soubeyran I 860
Soubeyran P 860
Soukos NS 261
Soutar D 580 (A)
Soutter P 554
Sozzi G 586
Spagnoli GC 1080
Sparen P 159
Author index 1433
British Journal of Cancer (1999) 81(8), 1429-1435
(c) Cancer Research Campaign 1999
Speirs V 690
Speizer FE 918
Spiegelman D 918
Spiess E 510
Sprangers MAG 87
Stahl M 1206
Stalla GK 381
Stamatis G 1206
Stamp GWH 431
Stampfer MJ 918
Stanson J 822
Statkevich P 1022
Steel CM 141
Steenbeek-Slotboom M 28
Stehle G 1428 (L)
Steinbuch M 900
Sten K 925
Stenback F 654
Stephens RW 203
Sterenborg HJCM 600
Stevens MCG 942
Stevenson D 1188
Stickeler E 179
Stott F 565 (A)
Strange R 572 (A)
Strange RC 571 (A), 1174
Strassburger S 1071
Stratford IJ 1127
Stuben G 1206
Stupnicka K 572 (A)
Stuschke M 1206
Sugase M 1234
Sugimori H 1234
Sugiyama T 95
Sullivan DC 583 (A)
Sumii K 647
Sumitomo M 277
Sun B 966
Sunami E 1274
Sutton R 581 (A)
Swann PF 583 (A)
Swanson CA 167
Swarts HGP 28
Swartz MA 756
Swensen AR 549
Szepeshazi K 966
Tachibana M 277
Tadir Y 631
Tagliaferri P 1134
Tahara E 647
Tajima T 1419
Tajima Y 350
Takahara J 769
Takahashi T 342
Takahashi Y 647
Takayanagi A 277
Takeda T 747
Takeya M 1009
Talieri M 1385
Tam NNC 1335
Tammilehto L 1111
Tampellini M 841
Tanaka M 721
Tanaka Y 108
Tang W-Y 237
Tang ZY 814
Tangoku A 469
Taniguchi K 1155
Tarragona A 572 (A)
Tavani A 890
Tavelin B 727
Taylor JC 783
Taylor Jr PT 75
Te Meerman GJ 1262
te Poele RH 1285
Teillac P 832
Tempfer C 855
Teppo L 1426
ter Haar NT 1410
Teren M 832
Terpstra OT 600
Terracciano L 1080
Terry G 554
Terzi A 1213
Teschler H 1206
Tesei A 609
Theis S 808
Thomas D 893
Thomson AH 99
Thornalley PJ 574 (A)
Thornton H 1427
Tian J 814
Tian XX 994
Tijssen CC 1150
Tisdale MJ 80
Tixi L 310
To TSS 994
Tokuda Y 1419
Tomioka T 350
Tomozawa S 1274
Tornielli S 586
Toshikuni N 1080
Townsend RR 1188
Traine G 846
Trangas T 1385
Traunecker HCL 942
Trere D 404
Tretli S 528
Tribukait B 1017
Trifiro M 850
Trojaneck B 1009
Tromberg BJ 631
Tsang KS 1122
Tsang YS 1122
Tsao SW 1335
Tsuji T 1080
Tsujimoto M 747
Tsujitani S 484
Tsuno N 1274
Tsunoda S 1155
Tsuruo T 1274
Tsutsumi Y 1155
Tucci E 1031
Turzynski A 790
Twelves C 99
Uchiyama A 721
Ueda Y 769
Uemura H 741
Ugnat A-M 152
Uhl E 381
Uhler RJ 167
Umesaki N 95
Umeshita K 747
Utermann G 242
Utoguchi N 1155
Vainio H 568 (A)
Vakkala M 592
van Beek J 114
van de Vijver MJ 1410
van den Berk PCM 43
van den Brule AJC 114
van den Ouweland AMW 1150
van der Graaf WTA 1262
van der Maas PJ 912
Van der Waal I 881
van Hille B 800
van Ineveld BM 912
Van Kooten M 846
van Muijen GNP 774, 1066
van Netten JP 1426 (L)
van Steenbrugge GJ 28
van Tellingen O 330
van Weerden WM 28
Vanhoefer U 1304
Vargas J 503
Varley JM 300
Vary CPH 1002
Vasey PA 99
Vaslamatzis M 69
Vaughan Hudson G 476
Veldhoen N 409
Veldman R 1328
Verbeek ALM 912
Verheijen RHM 114
Verkasalo PK 1426
Vermey M 934
Vesprini D 179
Vicente H 846
Viens P 449
Villavecchia G 1378
Villette J-M 832
Vincent C 1059
Virolainen MJ 1017
Vitale V 310
Vives C 571 (A)
Vogl FD 702
Voorhorst FJ 114
Vos CBJ 1410
Voss AC 80
Vousden K 565 (A)
Vrasdonk C 269
Waas ET 774
Wacholder S 175
Wagener C 870
Wagner E 580 (A)
Wakai Y 1155
Walboomers JMM 114, 881
Wallace HM 573 (A)
Wallen CA 925
Wallwiener D 225
Walton DS 690
Wang F 532
Wang GL 1122
Wang L-M 925
Wang TTY 387
Wang W 1182
Wang-Gohrke S 179
Ward BG 559
Warr JR 423
Warren R 1257
Watanabe T 1419
Watanabe Y 54
Watson PH 1002
Watson SA 19
Watterson J 409
Weber B 179
Weijer K 43
Weikel W 179
Weimar IS 43
Weinberg CR 900
Welby C 1188
Welker H-G 1269
Werle B 510
West CML 354
Wester K 1363
Westerveld A 1150
Westphal JR 774
Wever LDV 87
Wharton RQ 572 (A)
Wheatley D 573 (A)
Wheeler KT 925
White INH 577 (A)
White J 571 (A), 572 (A)
Whiteside TL 822
Whiting JL 1356
Whitman J 893
Wiechen K 790
Wiklund TA 1017
Wilbanks GD 582 (A)
Wilke H 1206
Wilks DP 354
Willett WC 918
Williams G 496
Williams R 431
Winfield D 476
Wittig B 1009
Wolf M 870, 1111
Wolff H 377
Wong N 850
Wong YC 1335
Wood SR 616
Wright EG 570 (A),
1095 (L)
Wu C-W 416
Wunder A 1428 (L)
Wyllie AH 141
Wyllie R 336
Xiao Z 638
Xie B 1335
Xu K 574 (A)
Xue Q 814
Yadav M 893
Yajima A 1234
Yakushiji M 95
Yamadori I 769
Yamaguchi M 712
Yamamoto K 469
Yamano T 1080
Yang C-H 796
Yang SC 127
Yano H 1274
Yarranton GT 972
Yasui W 647
Ye SL 814
Yokozaki H 647
Yoshida K 741
Yoshikawa H 1234
Yoshikawa K 741
Yotis J 1385
Young IE 141
Yu H 490
Yu MC 542
Yuan J-M 542
Yuan ZQ 850
1434 Author index
British Journal of Cancer (1999) 81(8), 1429-1435 (c) Cancer Research Campaign 1999
Zaffaroni N 252
Zander A 870
Zardi L 1071
Zeevi A 822
Zendman AJW 774
Zerah M 835
Zhang J 152
Zhang Q-X 672
Zoli W 609
Zori Comba A 846
Zubairi ST 581 (A)
Author index 1435
British Journal of Cancer (1999) 81(8), 1429-1435
(c) Cancer Research Campaign 1999

				

